# IgG4-related Disease Presenting as a Pancreatic Mass and Bilateral Lacrimal Gland Swelling

**DOI:** 10.7759/cureus.1054

**Published:** 2017-02-24

**Authors:** Uroosa Ibrahim, Amina Saqib, Nikhil Nalluri, Muhammad R Raza, Mark Goldstein

**Affiliations:** 1 Department of Hematology/Oncology, Staten Island University Hospital; 2 Pulmonary/Critical Care, Staten Island University Hospital; 3 Department of Internal Medicine, Staten Island University Hospital; 4 Cardiology, Staten Island University Hospital; 5 Rheumatology, Staten Island University Hospital

**Keywords:** igg4-related disease, rituximab, pancreatic mass, lacrimal gland, igg

## Abstract

IgG4-related disease is characterized by multi-system inflammation and possible elevation in serum immunoglobulin-G4 level. Treatment regimens include glucocorticoids, disease-modifying anti-rheumatic agents and recently, rituximab infusions have been reported to be effective in treatment-refractory disease. We present a case of a 64-year-old male presenting with acute abdominal pain and scleral icterus. An abdominal ultrasound demonstrated extensive biliary ductal dilatation. A computed tomography (CT) scan and a magnetic resonance cholangiopancreatography (MRCP) revealed a 4.8 cm pancreatic head mass. He underwent an exploratory laparotomy, and a pathologic examination of the mass revealed a dense lymphoplasmacytic infiltrate. The patient’s IgG subclass 2 level was elevated. A review of the patient’s medical records revealed that, in 1992, he presented with bilateral periorbital discomfort not severe enough to require intervention. In 2006, he presented with bilateral periorbital swelling and discomfort. A magnetic resonance imaging (MRI) scan showed gross enlargement of the lacrimal glands, and a biopsy revealed a dense lymphoplasmacytic infiltrate. He was treated with corticosteroids, cyclosporine and methotrexate. The regimen was repeated in 2009 for recurrent symptoms. Hence, on our encounter, a diagnosis of IgG4-related disease was made and he was treated with rituximab that resulted in complete remission.

## Introduction

IgG4-related disease is a multisystem disease recognized over the past few years. The name of the disease was coined at a research meeting in Japan in February 2010 [1]. The disease is characterized by an inflammatory process with a dense infiltrate of lymphocytes and plasma cells on histology and associated elevation in serum immunoglobulin G4 level. The precise mechanism of IgG4-related disease is unknown, and histological examination of the involved tissues is required for diagnosis. Presentation of symptoms may be acute, sub-acute, or chronic as described in our case. Treatment regimens include glucocorticoids and disease-modifying antirheumatic agents, such as azathioprine, cyclosporine, and methotrexate. Combinations are based on disease severity and individual response to therapy. Rituximab infusions have been reported to be effective in patients with treatment refractory disease [2].

Patient informed consent was obtained for this case report.

## Case presentation

**Figure 1:**
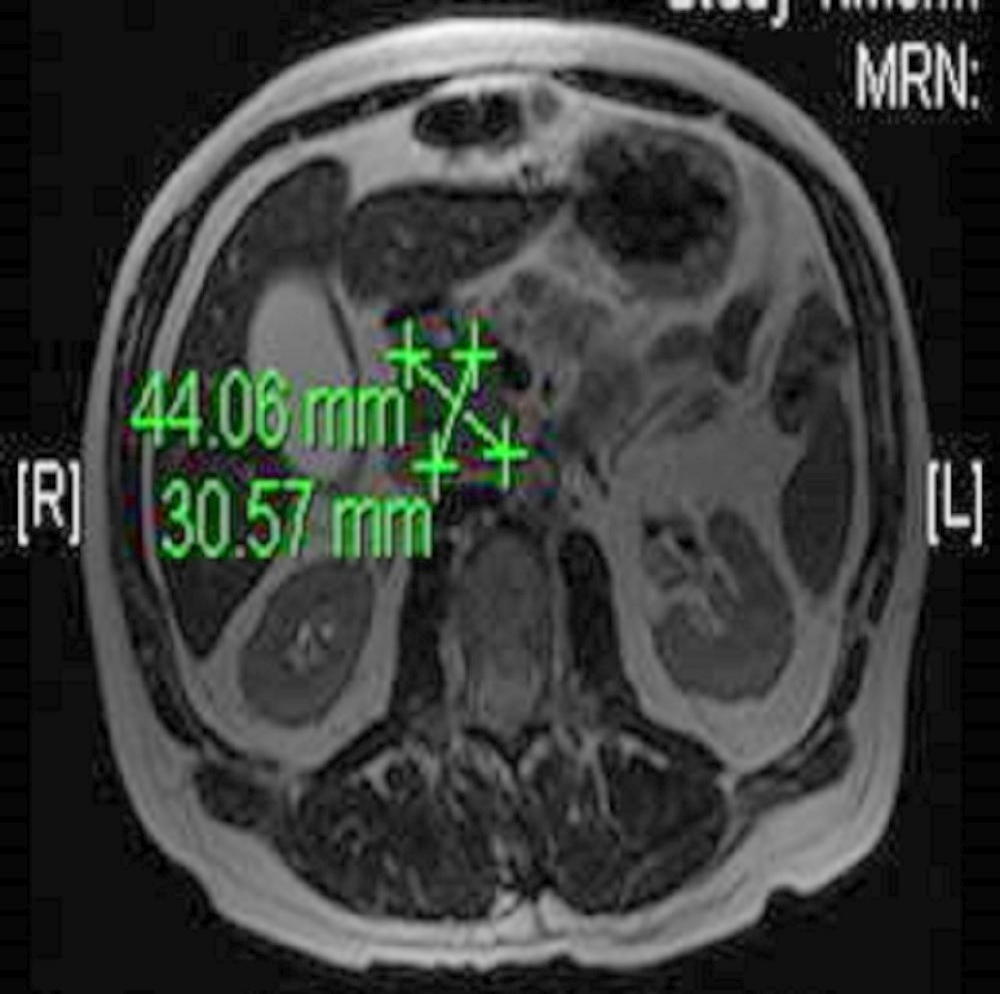
Magnetic resonance cholangiopancreatography (MRCP) A 4.4 x 3.1 x 4.8 cm mass in the region of the head of the pancreas is seen.

**Figure 2:**
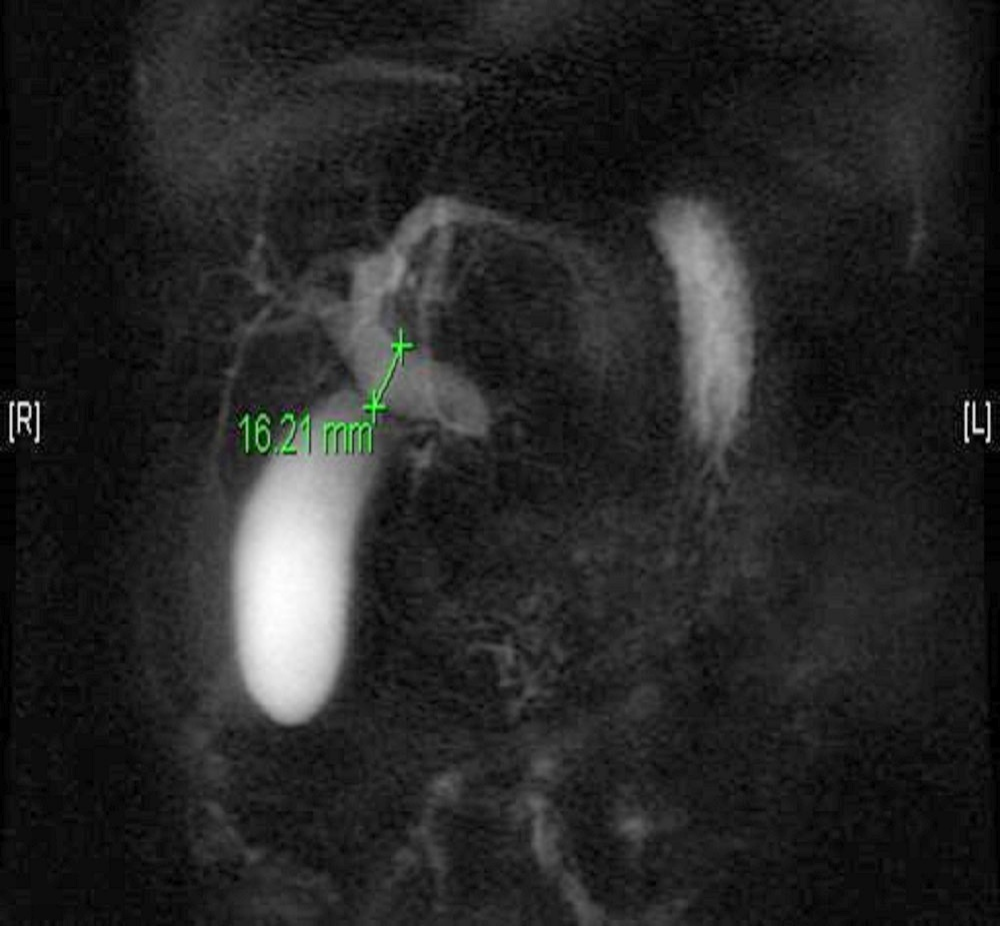
Magnetic resonance cholangiopancreatography (MRCP) Dilatation of the proximal bile duct to 1.6 cm is seen.

A 64-year-old male presented to the emergency department with the sudden onset of abdominal pain, night sweats, and decreased appetite. The patient’s past medical history included lichen planus, hypothyroidism, asthma, chronic sinusitis, and multiple allergies, including to pollen, molds, and shellfish. On admission, the total bilirubin was 2.0 mg/dL, alkaline phosphatase 454 IU/L, aspartate aminotransferase (AST) 252 IU/L, and alanine aminotransferase (ALT) 215 IU/L. Erythrocyte sedimentation rate was 83 mm/hr. Abdominal ultrasonography revealed dilatation of the intrahepatic and extrahepatic bile ducts and the presence of gallbladder sludge without evidence of gallbladder wall thickening, pericholecystic fluid, or cholelithiasis. A computed tomography scan revealed a 5 cm mass in the pancreatic head inseparable from the adjacent duodenum and moderate dilatation of the intrahepatic and extrahepatic biliary ducts with abrupt tapering of the common hepatic duct. Magnetic resonance cholangiopancreatography (MRCP) revealed the presence of a 4.4 x 3.1 x 4.8 cm mass in the head of the pancreas with a soft tissue filling defect in the distal common bile duct and dilatation of the proximal duct up to 1.6 cm [Figures 1-2]. No pancreatic ductal dilatation was present.

The following day, the patient's total bilirubin was up to 13.7 mg/dL with the direct fraction being 8.72 mg/dL. An endoscopic ultrasound-guided biopsy of the mass revealed necrotic tissue and the quantity was insufficient to reach a definitive pathological diagnosis. A percutaneous transhepatic cholangiogram was performed with placement of an external biliary drainage catheter. Considering the endoscopic ultrasound, computed tomography (CT) scan and MRCP findings, the presence of obstructive jaundice, and absence of evidence of metastatic disease, a decision to do a diagnostic laparoscopy and exploratory laparotomy with possible Whipple’s procedure and feeding tube jejunostomy was made. During the procedure, no evidence of ascites or carcinomatosis was observed. There was a mobile large mass in the head of the pancreas that extended into the neck, body, and part of the tail. It had the appearance of chronic pancreatitis. A frozen section obtained from the mass in the body of the pancreas showed inflammatory changes. No evidence of lymphoma or adenocarcinoma was found. Biopsies from the head of the pancreas were obtained for permanent section and the abdomen was closed uneventfully with a drain left in the left upper quadrant. Findings on gross examination of the specimen were significant for chronic pancreatitis with extensive sclerosis, acinar and ductal atrophy, and focal inflammation of venules causing luminal obliteration. The inflammatory infiltrate was a combination of lymphocytes and plasma cells. There was preservation of overall architecture with intact residual islets of Langerhans. Immunohistochemical staining revealed the presence of focally clustered numerous plasma cells. Further laboratory findings were significant for an IgG subclass 2 level of 1120 mg/dL (normal 241- 700 mg/dL), C4 complement of 49.2 mg/dL (normal 16-38 mg/dL), and C3 complement of 154.5 (normal 79-152 mg/dL). IgG subclass 1, 3, and 4 levels were normal.

A repeat contrast-enhanced CT scan done two days later revealed improvement in previously noted intrahepatic biliary ductal dilation, a small amount of peripancreatic fluid and the presence of a moderate amount of ascites. Resolution of the intrahepatic biliary ductal dilatation was observed on a cholangiogram performed twelve days later and the biliary catheter was removed. The patient’s liver function tests at this time were an alkaline phosphatase of 216 IU/L, AST 30 IU/L, ALT 36 IU/L, total bilirubin 1.5 mg/dL and direct bilirubin 0.83 mg/dL. Amylase and lipase were normal.

**Figure 3:**
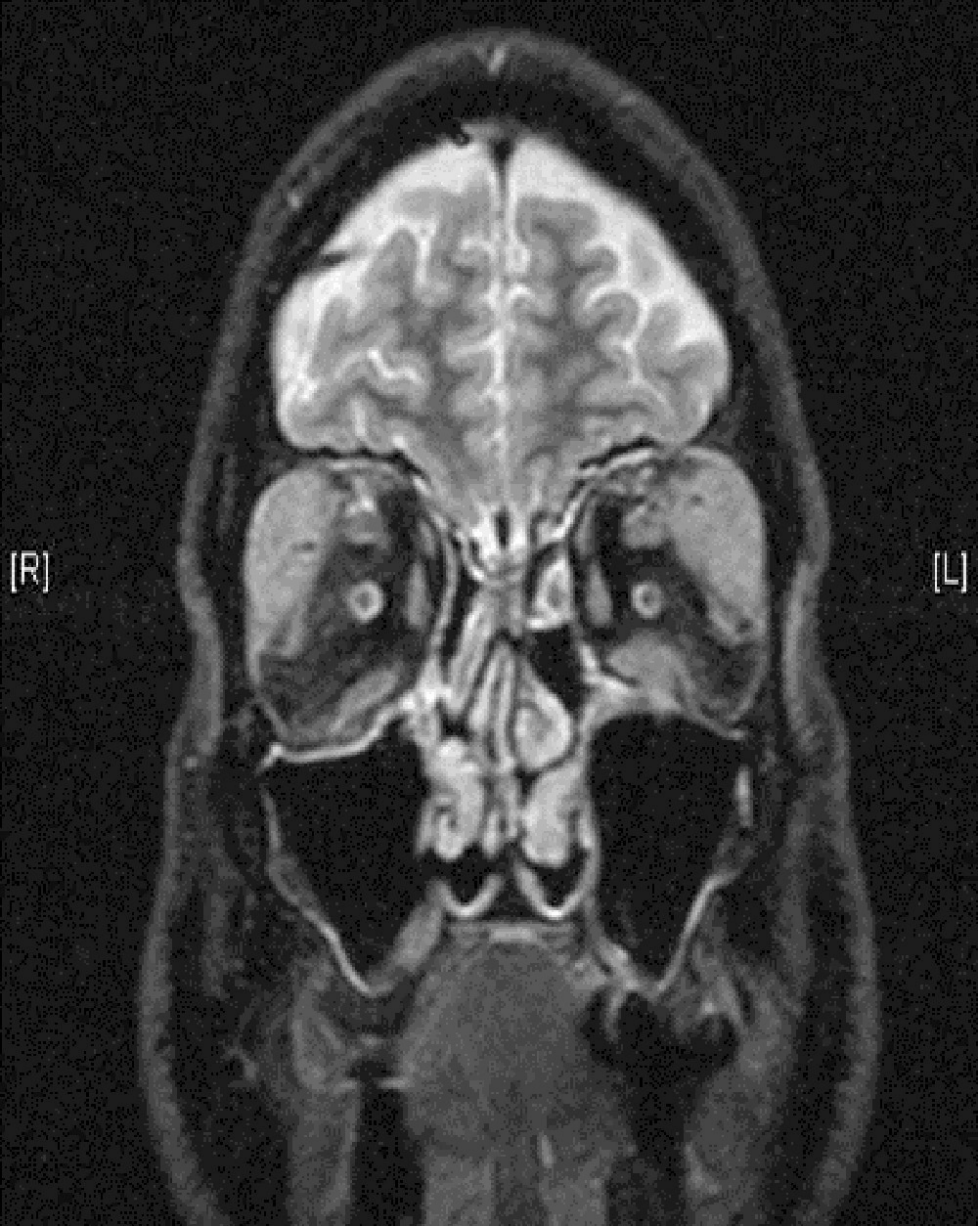
MRI Orbits Gross enlargement of the bilateral lacrimal glands with displacement of the extraocular muscles and globes is seen.

An extensive review of the patient’s past medical records revealed that in the year 1992, he had developed eye discomfort significant enough to seek medical attention. Magnetic resonance imaging (MRI) at the time revealed bilateral lacrimal gland enlargement with normal extraocular muscles, optic nerves, globes, and sinuses. The symptoms subsided without intervention. Fourteen years later, in 2006, the patient had noticed severe swelling around his eyes and had difficulty with upward gaze because of discomfort. Examination findings were pertinent for periorbital swelling and diminished upward gaze bilaterally. A CT scan revealed massively enlarged lacrimal glands extending posterior to the globes and wrapping around the globes on either side. MRI of the brain and orbits showed gross enlargement of the bilateral lacrimal glands and displacement of the extraocular muscles and globes (Figure 3). A biopsy of the periorbital tissue showed replacement of normal tissue with a heavy chronic inflammatory infiltrate with reactive lymphoid follicles and prominent germinal centers. A CD3 stain for T cells and a CD68 stain for histiocytes revealed a variable histiocytic infiltrate between the lymphoid follicles with occasional eosinophils and plasma cells. Based on the pathology findings, a diagnosis of xanthogranulomatous disease of the orbits was made. With the patient’s history of asthma and multiple allergies, he was categorized as having adult-onset asthma and periocular xanthogranuloma (AAPOX) syndrome [3]. The patient was treated with the following regimen: 1) three doses methylprednisolone 1 gram intravenously over two hours on alternate days followed by a three-month prednisone taper with 40 mg daily for one month, 30 mg daily for one month, then 10 mg daily for one month; 2) cyclosporine, 100 mg daily, for six months, then tapered off; and 3) methotrexate, 15 mg weekly for eight months, then tapered off. The patient had a substantial decrease in upper eyelid swelling by the end of the regimen. He reported no symptoms until three years later, in 2009, when he presented again with bilateral periorbital swelling, hyperacusis, and decreased extraocular movements. He was restarted on the previous drug regimen and this time methotrexate was tapered over eight months, and thereafter, he was kept on a maintenance dose of methotrexate of 7.5 mg weekly with folic acid. Meanwhile, he also started reporting symptoms of joint pain with morning stiffness lasting over one hour. There was no associated swelling, tenderness, or deformity. Rheumatologic disease workup was negative at the time. The patient remained asymptomatic until our encounter with him in 2011. He was relieved that malignancy had been ruled out and was discharged with a diagnosis of IgG4-related disease. He was treated with rituximab and remains in remission at the time of this publication.

## Discussion

IgG4-related disease is an emerging clinical entity characterized by multisystem inflammation with a dense lymphoplasmacytic infiltrate and possible elevated serum IgG4 levels. Data pertaining to the epidemiology of the disease come from Japan and are estimated from the prevalence of autoimmune pancreatitis which is noted to be 0.8 per 100,000 persons in Japan [4]. The name of the disease was coined at a research meeting in Kanazawa, Japan in February 2010 [1]. Involvement of multiple sites has been described, including lacrimal and submandibular glands, thyroid and pituitary gland, pancreas, kidneys, and the retroperitoneum. The disease is noted to have a male predominance and is more common above 50 years of age [5]. The clinical manifestations depend on the organ system involved and may be acute, subacute, or chronic. As seen in our patient, he had subclinical swelling of periorbital tissue not requiring urgent attention for up to 12 years. Thereafter, the ocular discomfort made him seek intervention at which time it may be said to be subacute. However, five years later, he presented acutely with pancreatic involvement requiring urgent attention. Hence, our case is unique in terms of presenting with chronic, subacute, and acute findings over a period of time.

Allergic disease and tumefactive lesions are observed as common findings in patients with IgG4-related disease [6]. Both also were present in our patient. Tissue biopsy with histopathological examination and immunohistochemical staining are essential and are the gold standard for diagnosis. Elevated serum IgG4 levels may or may not be present and are not required for definite diagnosis. Histology findings in favor of IgG4-related disease include a dense lymphoplasmacytic infiltrate with IgG4 positive plasma cells. In glandular organs, there is sparing of ductal structures with periductal sclerosis. Obliterative phlebitis may also be present. On immunohistochemical staining, plasma cells positive for IgG4 are seen. Imaging studies may be required depending on symptoms, although findings may not help distinguish IgG4-related disease from malignancy. However, they help delineate lesions and provide guidance for biopsy [7].

Treatment of IgG4-related disease depends on acuity of symptom onset. Asymptomatic lymphadenopathy has been noted and observed inconsequentially for decades [8]. However, certain manifestations require urgent attention, such as in our patient, watchful waiting was sufficient for up to 12 years when he had subclinical periorbital swelling. Increasing discomfort required intervention with biopsy and medications. Involvement of the pancreas had him seek urgent attention, hospitalization, and extensive workup, eventually reaching a diagnosis of IgG4-related disease. First line treatment of IgG4-related disease involves glucocorticoids. Various regimens ranging from three months to three years have been used [9]. However, relapses are common. Azathioprine, methotrexate, and cyclosporine can be used as steroid-sparing drugs or as maintenance therapy. The immune modulator, rituximab, can be used in patients with recurrent or treatment refractory disease. Rituximab is a chimeric monoclonal antibody targeting CD20 protein on the surface of B cells. It results in B-cell depletion and a decline in IgG4 level. It has been hypothesized that rituximab prevents B-cell maturation into plasma cells; however, the precise mechanism leading to decrease in IgG4 concentration is not known [10].

## Conclusions

IgG4-related disease should be suspected in patients presenting with multisystem inflammatory involvement. A high index of suspicion is required in patients presenting with tumorous growth in various sites, including lacrimal glands, salivary glands, and pancreas. Our case provides insight into how a suspected diagnosis of cancer turned out to be a benign disease. It also provides a spatial and temporal outline of the course of this disease entity and its response to various treatment modalities.
